# Accuracy and validation of a point-of-care blood glucose monitoring system for use in horses

**DOI:** 10.3389/fvets.2024.1436714

**Published:** 2024-10-10

**Authors:** Sridhar Velineni, Paul Schiltz, Ko-Hsin Chang, Yi-Ming Peng, Bobby Cowles

**Affiliations:** ^1^Veterinary Medicine Research and Development, Zoetis, Kalamazoo, MI, United States; ^2^Associate Professor and Director of Equine Studies, William Woods University, Fulton, MO, United States; ^3^Tyson Bioresearch Inc., Zhubei City, Taiwan; ^4^Equine Technical Services, Zoetis, Parsippany, NJ, United States

**Keywords:** AlphaTrak 3, blood glucose monitoring, equine, hypoglycemia, hyperglycemia, glucometer, point-of-care, ISO15197:2013

## Abstract

Abnormal blood glucose (BG) levels often seen in critically ill horses are significantly associated with adverse patient outcomes and increased mortality. Rapid and accurate BG monitoring is now considered an essential component of evidence-based equine practice and can provide critical information quickly for treatment. Although several point-of-care (POC) BG monitoring hand-held devices are commercially available for veterinary use, none contains a unique algorithm validated for use in horses. The AlphaTrak 3 (AT3) BG monitoring system is a first-of-its-kind device with an equine-specific algorithm that allows stall-side clinical decision making, and frequent monitoring at minimal cost. As such, AT3 is potentially a preferred alternative to more costly and time-consuming standard diagnostic reference laboratory methods. The objective of this study was to determine the accuracy of the AT3 device in measuring BG levels in equine whole blood samples in comparison to results obtained by the Beckman Coulter AU480 reference analyzer per ISO15197:2013 specifications. Accuracy of the AT3 equine algorithm were initially verified by testing equine blood samples with artificially adjusted blood glucose levels followed by its validation in a field study. Testing with artificially adjusted equine samples (*n* = 129) showed that 98.9% of glucose measurements ranging from 29 to 479 mg/dL fell within ISO accuracy threshold of ±15 mg/dL or ±15% of the average reference value. In addition, 100% of the AT3 measurements fell in consensus error grid (CEG) zone A, which indicates that test outcomes have a minimal likelihood of adverse clinical impact. In a follow-up field study involving 96 horses, 98.4% of AT3 measurements met the ISO accuracy threshold and 99.2% of AT3 measurements fell in CEG zone A. These results demonstrate that the AT3 glucometer has a high degree of accuracy in horses and is a dependable, convenient, and cost-effective device for accurately monitoring equine BG levels in farm or clinical settings.

## 1 Introduction

Blood glucose (BG) is tightly regulated in healthy horses, with normal reference ranges variously established at 62–134 mg/mL (3.4–7.4 mm/L), 76–131 mg/dL (4.2–7.3 mm/L), and 70–135 mg/dL (3.9–7.4 mm/L) depending on the source (1, 2). Hypoglycemia, hyperglycemia and glucose variability, on the other hand, are associated with a variety of morbidities in horses. For example, prior studies found that septic and critically ill foals with relatively low or hypoglycemic BG concentrations at presentation had significantly reduced survival rates from birth to 96 h compared to foals with higher BG levels (3, 4). A study of critically ill neonatal foals (*n* = 515) presented at veterinary school hospitals found that >70% were either hypoglycemic (34.4%) or hyperglycemic (36.5%) at the time of admission (5). Glucose dysregulation in horses is known to be associated with acute gastrointestinal disease and suboptimal survival rates (1, 2). In one study, >50% of horses (*n* = 269) presenting with acute abdominal disease were hyperglycemic and had significantly reduced short-term (*p* < 0.05) and long-term (*p* < 0.016) survivability (2).

A discordant BG level by itself may not have been the primary cause of increased mortality in these studies. However, the authors collectively concluded that BG appears to be a strong and readily accessible surrogate indicator of various biological factors that contribute to equine mortality (1, 2). It is apparent that the extent and duration of equine BG concentrations outside the normal range are significantly associated with adverse patient outcomes, poor prognosis, reduced post-diagnosis duration of survival, and increased mortality.

As the role of BG dynamics in equine homeostasis and disease etiology is becoming better understood, rapid and accurate methods of glucose monitoring are considered an essential component of evidence-based equine practice. Particularly in critical care settings, serial on-site BG monitoring enables veterinarians to promptly identify hypo- and hyperglycemia in horses and initiate rapid intervention, including glucose regulation and other treatment protocols. The need for aggressive BG monitoring is increasingly relying on point-of-care (POC), stall-side glucometers as a preferred alternative to more costly and less timely analysis by diagnostic reference laboratories.

Portable, hand-held POC glucometers are the most commonly used devices for BG monitoring in clinical practice and at-home (6, 7). While portable POC glucometers have been in widespread use for diabetes management in human healthcare since the 1970s (8), these devices have been adopted more gradually in veterinary medicine. In the past two decades, studies have evaluated the accuracy of POC glucometers in cats, dogs, calves, laboratory animals, and non-human primates, with varying degrees of diagnostic accuracy (8–12). Particularly in equine critical-care cases where BG dysregulation is associated with increased mortality (1–4), the importance of accurate and prompt BG monitoring is being increasingly recognized. In horses, accuracy of portable POC glucometers has been reported in numerous studies in the U.S., Europe, and Australia (5, 9, 11, 13–19).

In most cases, POC glucometers evaluated in animals were originally developed for use in humans rather than in domestic animal species (14). However, expert opinion suggests that human glucometers should be avoided for measuring BG levels in animals because of the lack of equivalence in interspecies glucose distribution (14, 20). For example, glucose distribution in humans is closely divided between plasma (58%) and erythrocytes (42%), while plasma glucose distribution is 93% in cats, 87.5% in dogs, and 84% in rats (8, 14). Others have noted that species-specific pathophysiology may create bias in BG concentration (15), and that species differences in erythrocyte morphology and size may affect erythrocyte glucose concentration (8, 10). For these reasons, current canine and feline diabetes management guidelines go so far as to recommend against using human glucometers in dogs and cats (20). The preferred alternative is to use POC glucometers that have been specifically validated for each species in which they are used.

The on-market AlphaTrak 2 (AT2) BG monitoring system (Zoetis, Parsippany, NJ, USA) is an accurate, easy-to-use, handheld POC glucometer that requires a small sample volume (≥0.3 μL) to provide results in seconds, and developed for veterinary use, specifically for dogs and cats. AlphaTrak 3 (AT3) is a next-generation POC glucometer with applicability both in clinical and on-farm settings, with online connectivity capability and a first-of-its-kind equine-specific algorithm. The ability to accurately measure BG quickly will allow for early intervention to correct the issue and potentially improve outcomes in patients. The objective of this study was to determine the accuracy of the AT3 glucometer in measuring BG levels in horses using an equine algorithm and validating the AT3 results by comparing them with BG values obtained by a reference laboratory method.

## 2 Materials and methods

### 2.1 AlphaTrak 3 blood glucose monitoring system

AlphaTrak 3 (Zoetis, Parsippany, NJ, USA) is a veterinary BG monitoring system, which can selectively use algorithms for cats and dogs. This POC device can measure glucose levels from capillary or EDTA or heparin anti-coagulated venous whole blood (WB) sample volumes ≥0.3 μL with hematocrit levels ranging between 15 and 65%. A lancing device with single-use replaceable lancets included as part of the AT3 system ensures the safe collection of the capillary blood required to perform a test. When a test strip (TS) for capturing the blood sample is inserted into this glucometer, the device is activated. Following application of a blood sample, the TS draws the sample into the sample-receiving chamber for analysis. Accurate results for BG levels ranging from 20 to 750 mg/dL (1.1–41.7 mmol/L) are typically displayed within 5 min. The system includes Bluetooth-enabled, web-based, and mobile applications that facilitate the sharing, storing and transfer of test results via the internet to a veterinary clinic. The AT3 mobile app can also programmed to receive reminders for follow-up tests.

### 2.2 Accuracy of AT3 system in measuring artificially adjusted equine blood samples

Venous WB samples derived from 60 different horses (31 male and 29 females, age, 3–22 years, hematocrit levels, 23–40%) in vacutainer heparin tubes were utilized for this study. Following equilibration of the blood sample to room temperature, the basal level glucose concentration of each sample was measured on the YSI 2900 Biochemistry Analyzer per manufacturer’s instructions by separating plasma by centrifugation at 3000 rpm for 5 min. The WB samples were artificially adjusted to generate a set of 129 blood samples with different glucose levels ranging from 29 to 479 mg/dL, which included (1) 60 unaltered blood samples in the normal glucose range, (2) 60 samples spiked with 20% condensed glucose solution to generate samples in the hyperglycemic range, and (3) 9 samples incubated at 37°C for ≤24 h to deplete their glucose levels to the hypoglycemic range. Glucose concentration of a given blood sample was measured using 2 test strips per each of three different lots on six AT3 devices. At the end of testing on the AT3 devices, leftover blood from the same sample were centrifuged immediately to separate plasma, which were stored at −80°C until shipped for reference testing. The same operator sequentially repeated the above testing protocol with each of the remaining blood samples with different glucose levels on AT3 devices generating a total of 774 measurements.

### 2.3 Validation of an equine-specific algorithm in a field study

As the prevalence of hypoglycemia is common in foals, the accuracy of the AT3 equine algorithm in measuring hypoglycemic equine samples was further validated in a field study involving 96 horses that belong to 18 different breeds (Arabian, American saddlebred, Thoroughbred, Quarter horse) of various ages (4–25 years). All horses were maintained at an equine farm at William Woods University, Fulton, Missouri. Venous WB samples collected in two vacutainer EDTA tubes (approximately 3.0 mL each) from each horse. An attending veterinarian supervised collection of equine venous blood samples and BG testing using the AT3 system. Immediately after collection, BG concentration of a given sample in tube 1 was concurrently measured in duplicate using three TS lots on three AT3 meters. Within 10 min after testing on AT3 devices, the leftover sample in tube 1 was centrifuged, and the plasma fraction was stored at −20°C until shipped for reference testing. Blood samples in tube 2 were subjected to glycolysis at 37°C for 1 h to deplete BG concentrations to a hypoglycemic range of approximately 66–105 mg/dL and tested on AT3 devices as described above using three strip lots. Immediately following AT3 testing, plasma was separated from the leftover glucose depleted sample in tube 2 by centrifugation and stored at −20°C until shipped for reference testing.

Testing of remaining WB samples and their corresponding glucose depleted samples was performed on the AT3 devices as described above. All plasma samples from both sets of collection tubes (fresh blood and glucose depleted) were then submitted for reference glucose testing. At the conclusion of the study, the AT3 testing data were submitted for biometric analyses.

At the end of each day of testing, all plasma samples were shipped overnight on ice to a Zoetis reference laboratory (San Diego, CA, USA) to measure glucose concentration on the reference Beckman Coulter AU480 Biochemistry Analyzer in triplicate.

### 2.4 Data analyses and acceptance criteria

ISO 15197:2013 guidelines were used to assess the accuracy of the AT3 device as compared to Beckman Coulter AU480 Chemistry analyzer reference measurements. The ISO 15197:2013 criteria stipulate that (1) 99% of all results are required to be in Zones A and B of the CEG, and (2) at glucose concentrations <100 mg/dL (< 5.55 mmol/L), 95% of the test results are required to be within ±15 mg/dL (±0.83 mmol/L) of the average reference value, and at glucose concentrations ≥100 mg/dL (≥5.55 mmol/L), 95% of the test results are required to be within ±15% of the average reference value. Clinical relevance of the AT3 system measurements were analyzed by Consensus Error Grid (CEG) distribution (21, 22). The CEG process is divided into five zones, which are defined by estimated risk to the animal if a result falls within a respective zone. Zones A and B indicate little or no adverse clinical effect of the test outcome. The deviation (bias) of BG values measured by AT3 versus the values measured by the reference analyzer were calculated. Bias plots displayed the BG values measured by AT3 in comparison to the average reference (6).

## 3 Results

**Figure 1:**
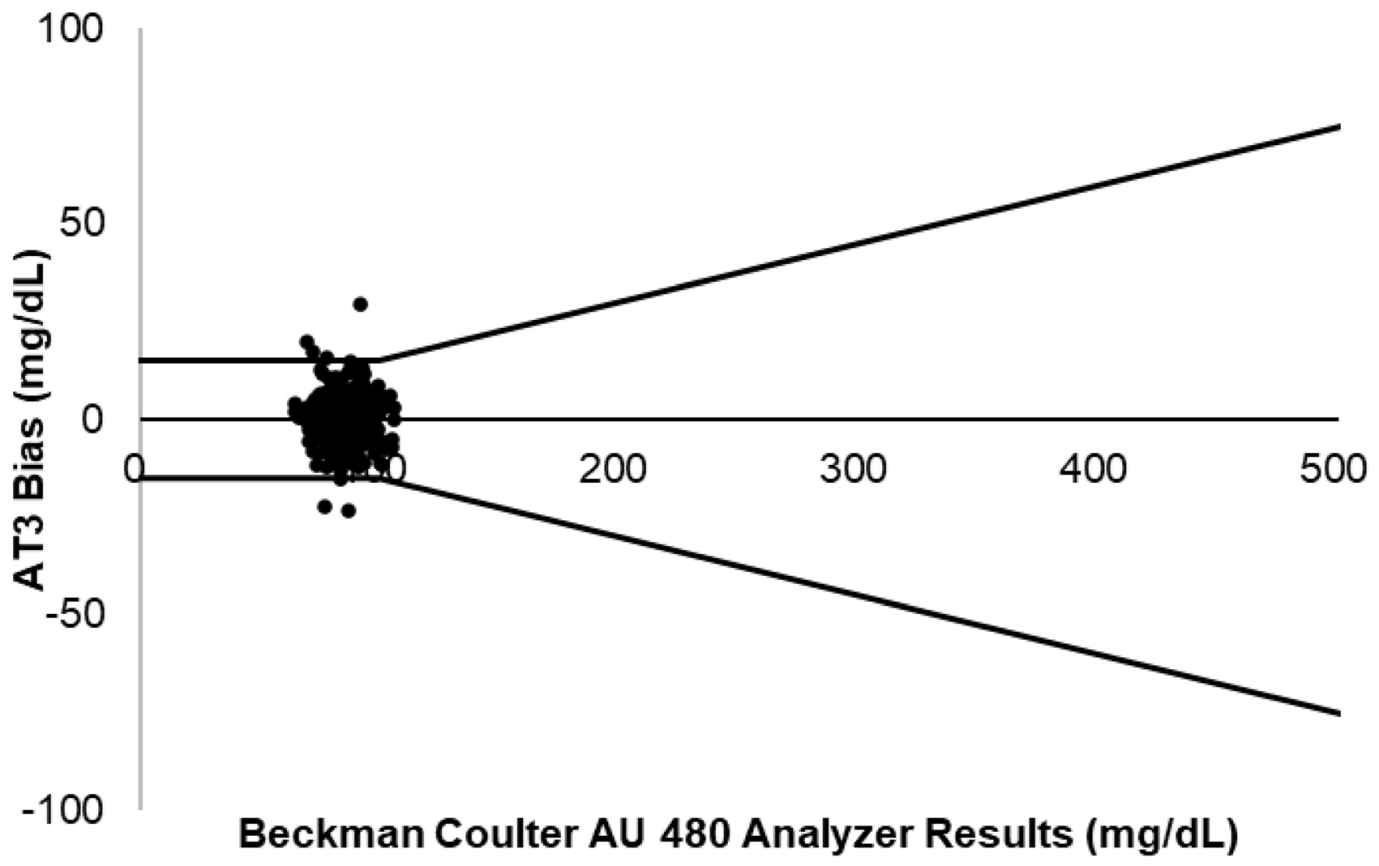
Bias plots for AT3 blood glucose results versus the Beckman Coulter AU480 laboratory reference method when tested with artificially adjusted equine blood samples (258 measurements per test strip lot). Upper and lower lines mark the upper and lower limits of ISO15197:2013 accuracy criteria, respectively.

**Figure 2:**
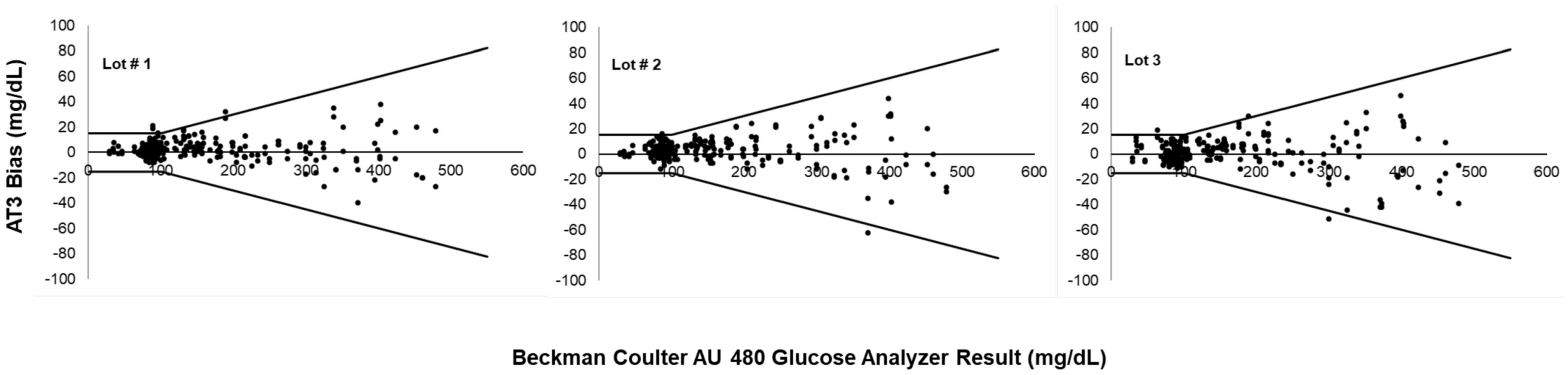
Consensus error grid (CEG) analysis of the AT3 system when tested with artificially adjusted equine blood samples (258 measurements per test strip lot).

At the BG concentrations tested in this study, 98.96% (766 out of 774) of AT3 measurements fell within the ISO accuracy limits when analyzed with the equine algorithm for all three strip lots. A small subset 1.04% (8 out of 774 measurements) of equine measurements fell outside ISO accuracy threshold. Results confirm that accuracy of the AT3 system were maintained across a broad range of BG values (29–479 mg/dL) including equine blood samples with artificially adjusted BG levels. Bias plots of the AT3 accuracy study test results versus the laboratory reference standard show that all three TS lots provided a high degree of compliance with the ISO standard, and that all three TS lots produced comparable accuracy results (Figure 1). Out of 258 AT3 measurements per each of three TS lots, (three different production runs), the number of AT3 measurements fell outside the reference range were 3, 2, and 3, respectively. Consensus error grid plots for all three strip lots illustrate that 100% of BG test values fell within CEG zone A, indicating that deviation of AT3 test results from the reference standard represented no adverse clinical risk (Figure 2).

**Figure 3:**
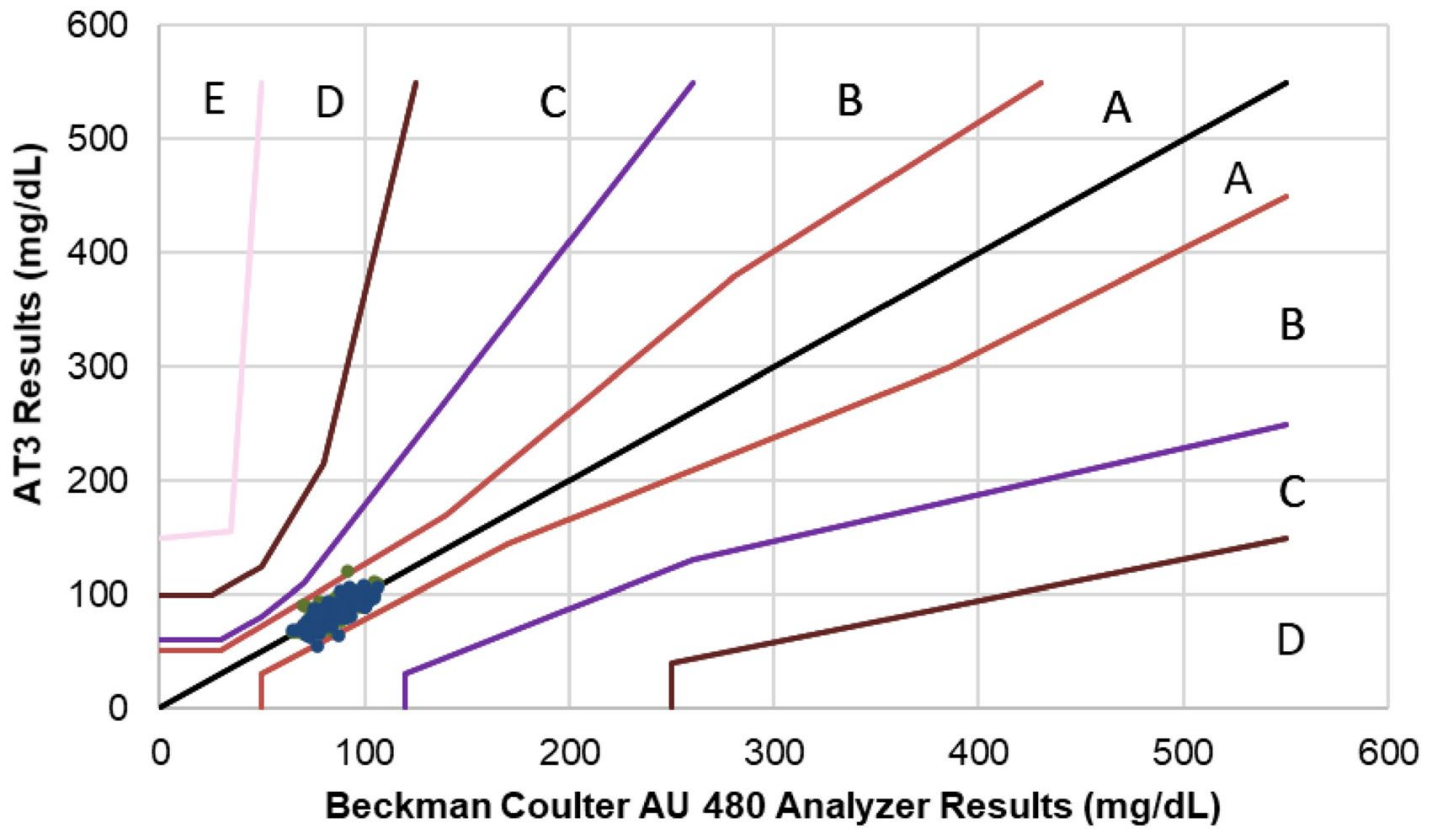
Bias plots for AT3 blood glucose results versus the Beckman Coulter AU480 laboratory reference method measured in the field study.

**Figure 4:**
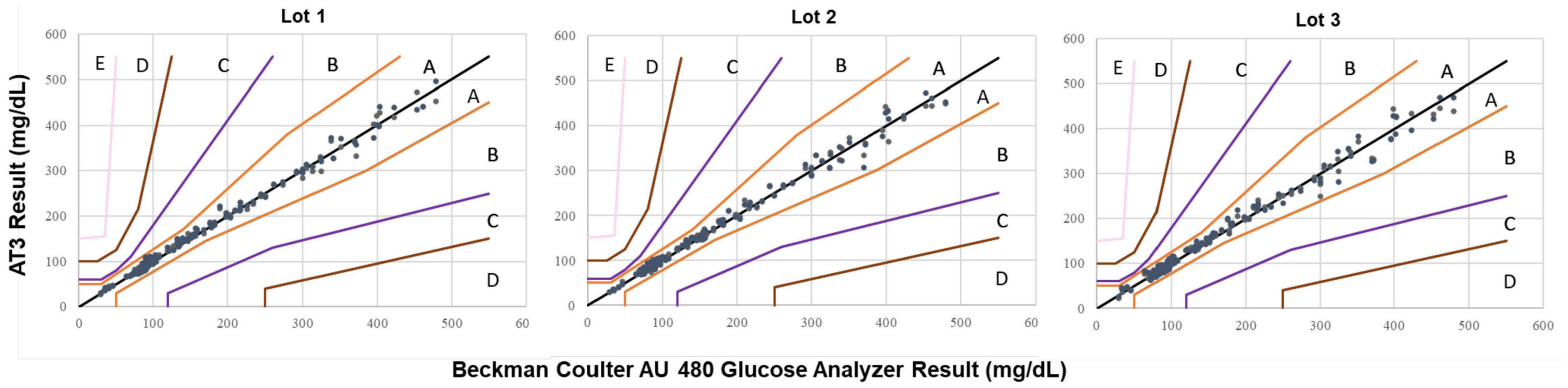
Consensus error grid (CEG) analysis of the AT3 measurements measured in the field study.

The accuracy of the AT3 equine algorithm in measuring hypoglycemic equine samples were further validated in a field study. Whole blood samples from 96 horses, including a subset of corresponding 87 glucose depleted samples, provided a total of 183 samples with BG levels ranging from 65 to 105 mg/dL for analysis. Testing 183 equine blood samples on duplicate test strips generated a total of 365 glucose measurements (one sample measurement was recorded incorrectly and eliminated from the analysis). Of the 365 AT3 measurements, 98.3% (345/351) of measurements at glucose concentrations <100 mg/dL were within the ISO accuracy threshold of ±15 mg/dL of the average reference value (Table 1). Additionally, 100.0% (14/14) of measurements at glucose concentrations ≥100 mg/dL were within the ±15% of the average reference value. The bias plot for the field study shows that all BG values measured by the AT3 system were within the ISO accuracy limits (Figure 3). A CEG plot for (Figure 4) shows that 99.2% (362/365) of BG samples measured by the AT3 system were within zone A and the remaining 3 samples were within zone B (Figure 4), thus meeting ISO criteria.

**Table 1:** Accuracy of AlphaTrak 3 blood glucose monitoring system in measuring equine glucose levels in a field study.

Host BG level	AT3 system accuracy results		
	Within ± 5 mg/dL or ± 5%	Within ± 10 mg/dL or ± 10%	Within ± 15 mg/dL or ± 15%
<100 mg/dL	65.8% (231/351)	90.9% (319/351)	98.3% (345/351)
≥100 mg/dL	28.6% (4/14)	85.7% (12/14)	100.0% (14/14)
All samples	64.4% (235/365)	90.7% (331/365)	98.4% (359/365)

## 4 Discussion

The AT2 system were developed for veterinary use with validated algorithms for accurate measurements of glucose levels in canine and feline blood samples. However, the AT2 feline algorithm was determined to be appropriate in testing equine blood samples. When used in horses (*n* = 50) and foals (*n* = 50) in an earlier study, the AT2 system had a median bias of 6.1% in adult horses and 5.0% in foals when BG levels were compared to Hitachi 917 Blood Chemistry System results, and 97% of BG values were within CEG zone A (14). The AT3 system is the next generation version of AT2 glucometer with connectivity capability and a first-of-its-kind equine-specific algorithm.

The principal outcome of this study was the confirmation that equine algorithm used in the AT3 system was well within the ISO 15197:2013 accuracy limits when compared to the BG concentrations measured by Beckman Coulter AU480 analyzer and YSI 2900 analyzer (unpublished data). Overall, at the glucose concentrations tested in this study, >98% of AT3 measurements fell within the ISO accuracy limits when analyzed with the equine algorithm for three different strip lots. Additionally, both AT3 feline and canine algorithms performed equivalently in measuring equine blood samples, but their accuracy was slightly lower as compared to the performance of AT3 equine algorithm (unpublished data).

As a measure of glucometer performance, the CEG was developed as an alternative to a straightforward bias percentage of BG samples that meet the ISO standard (21, 22). Each of the five CEG zones represents a degree of clinical risk posed by the BG test deviation from the reference method, progressing from zone A (accurate measurement with no effect on clinical outcome) to zone E (measurement error could have dangerous consequences). The AT# device, with the equine algorithm, performed reliably for reporting BG concentrations. In this study, 100.0% of AT3 measurements were within CEG zones A and B, indicating that any AT3 measurement deviation from the reference standard pose little or no clinical risk to the horse. Zone B indicates benign measurement error with little or no effect on clinical outcome (6, 21, 22).

Although nearly all horses in this study had BG values in zone A of the CEG, individual deviation from the reference standard tended to increase as BG levels rose (Figure 1). This was consistent with results of other studies measuring BG levels and glucometer performance in various species (6, 9, 10, 15, 17, 23). Previous studies have reported variations in results for different lots of test strips in the same glucometer and even when different glucometers of the same model using test strips from the same lot are compared (24, 25). In our study, analysis of each BG sample using multiple TS lots and AT3 glucometers helped negate the effects that any variations in testing system components may have had on assay results. No significant TS lot-to-lot variability were observed (unpublished data). In clinical practice, hematocrit values outside the normal range can also affect the accuracy of glucometer results (10, 26). While the high degree of diagnostic accuracy of the AT3 device in horses is encouraging, it is still good practice to confirm POC glucometer results that are aberrant, inconsistent with clinical findings, or highly variable for the same patient from one test to the next by reassessing the outcomes by means of laboratory analysis (18).

Collectively, the accuracy bias and CEG results from our study affirm that the AT3 system has a high degree of accuracy for BG measurements in horses. While laboratory assays remain the gold standard for BG measurement, this approach is impractical for serial monitoring of BG levels in-clinic or on-farm settings. Ease of use, a wide BG dynamic range, rapid determination of assay results, the need for only ≤0.3 μL blood sample, relatively low cost, and online connectivity represent advantages of the AT3 device versus laboratory assays.

The AT3 glucometer with an equine-specific algorithm and connectivity capability is an improvement over its predecessor AT2 system and represents a reliable and cost-effective method for accurately monitoring BG levels in horses on-farm, in-clinic, or laboratory settings to better address patient needs in a timely manner.

## Data Availability

The raw data supporting the conclusions of this article will be made available by the authors, without undue reservation.

## References

[1] Hassel DM Hill AE Rorabeck RA. Association between hyperglycemia and survival in 228 horses with acute gastrointestinal disease. J Vet Intern Med. (2009) 23:1261–5. doi: 10.1111/j.1939-1676.2009.0395.x 19780927

[2] Hollis AR Boston RC Corley KT. Blood glucose in horses with acute abdominal disease. J Vet Intern Med. (2007) 21:1099–103. doi: 10.1111/j.1939-1676.2007.tb03070.x, PMID: 17939570

[3] Gayle JM Cohen ND Chaffin MK. Factors associated with survival in septicemic foals: 65 cases (1988-1995). J Vet Intern Med. (1998) 12:140–6. doi: 10.1111/j.1939-1676.1998.tb02109.x, PMID: 9595374

[4] Peek SF Semrad S McGuirk SM Riseberg A Slack JA Marques F. Prognostic value of clinicopathologic variables obtained at admission and effect of antiendotoxin plasma on survival in septic and critically ill foals. J Vet Intern Med. (2006) 20:569–74. doi: 10.1111/j.1939-1676.2006.tb02898.x, PMID: 16734091

[5] Hollis AR Furr MO Magdesian KG Axon JE Ludlow V Boston RC. Blood glucose concentrations in critically ill neonatal foals. J Vet Intern Med. (2008) 22:1223–7. doi: 10.1111/j.1939-1676.2008.0174.x 18691362

[6] Freckmann G Pleus S Grady M Setford S Levy B. Measures of accuracy for continuous glucose monitoring and blood glucose monitoring devices. J Diabetes Sci Technol. (2019) 13:575–83. doi: 10.1177/1932296818812062, PMID: 30453761 PMC6501529

[7] Girardin CM Huot C Gonthier M Delvin E. Continuous glucose monitoring: a review of biochemical perspectives and clinical use in type 1 diabetes. Clin Biochem. (2009) 42:136–42. doi: 10.1016/j.clinbiochem.2008.09.112, PMID: 18951887

[8] Morley LA Gomez TH Goldman JL Flores R Robinson MA. Accuracy of 5 point-of-care glucometers in C57BL/6J mice. J Am Assoc Lab Anim Sci. (2018) 57:44–50. PMID: 29402351 PMC5875097

[9] Johnson PJ Wiedmeyer CE LaCarrubba A Messer NT Dingfelder HA Cogswell AM. Clinical assessment of blood glucose homeostasis in horses: comparison of a continuous glucose monitoring system with a combined intravenous glucose and insulin test protocol. J Vet Intern Med. (2011) 25:162–5. doi: 10.1111/j.1939-1676.2010.0643.x 21223373

[10] Karapinar T Tumer KC Buczinski S. Evaluation of the freestyle Optium Neo H point-of-care device for measuring blood glucose concentrations in sick calves. J Vet Intern Med. (2020) 34:1650–6. doi: 10.1111/jvim.15794, PMID: 32420677 PMC7379022

[11] Wiedmeyer CE Johnson PJ Cohn LA Meadows RL. Evaluation of a continuous glucose monitoring system for use in dogs, cats, and horses. J Am Vet Med Assoc. (2003) 223:987–92. doi: 10.2460/javma.2003.223.987, PMID: 14552487

[12] Zini E Moretti S Tschuor F Reusch CE. Evaluation of a new portable glucose meter designed for the use in cats. Schweiz Arch Tierheilkd. (2009) 151:448–51. doi: 10.1024/0036-7281.151.9.448, PMID: 19722134

[13] Cunneen A Wood KA Mathison K Herndon AM Bertin FR. Comparison of a continuous indwelling glucometer with a point-of-care device in healthy adult horses. Vet Rec. (2020) 187:e21. doi: 10.1136/vr.105607 32179578

[14] Hackett ES McCue PM. Evaluation of a veterinary glucometer for use in horses. J Vet Intern Med. (2010) 24:617–21. doi: 10.1111/j.1939-1676.2010.0481.x, PMID: 20337908

[15] Hollis AR Dallap Schaer BL Boston RC Wilkins PA. Comparison of the Accu-Chek Aviva point-of-care glucometer with blood gas and laboratory methods of analysis of glucose measurement in equine emergency patients. J Vet Intern Med. (2008) 22:1189–95. doi: 10.1111/j.1939-1676.2008.0148.x, PMID: 18638018

[16] Hug SA Riond B Schwarzwald CC. Evaluation of a continuous glucose monitoring system compared with an in-house standard laboratory assay and a handheld point-of-care glucometer in critically ill neonatal foals. J Vet Emerg Crit Care. (2013) 23:408–15. doi: 10.1111/vec.12072, PMID: 23859299

[17] Malik CE Wong DM Dembek KA Wilson KE. Comparison of two glucose-monitoring systems for use in horses. Am J Vet Res. (2022) 83:222–8. doi: 10.2460/ajvr.21.05.0068, PMID: 35038307

[18] Russell C Palmer JE Boston RC Wilkins PA. Agreement between point-of-care glucometry, blood gas and laboratory-based measurement of glucose in an equine neonatal intensive care unit. J Vet Emerg Crit Care. (2007) 17:236–42. doi: 10.1111/j.1476-4431.2007.00236.x

[19] Vitale V Berg LC Larsen BB Hannesdottir A Dybdahl Thomsen P Laursen SH. Blood glucose and subcutaneous continuous glucose monitoring in critically ill horses: a pilot study. PLoS One. (2021) 16:e0247561. doi: 10.1371/journal.pone.0247561, PMID: 33626099 PMC7904136

[20] Behrend E Holford A Lathan P Rucinsky R Schulman R. 2018 AAHA diabetes management guidelines for dogs and cats. J Am Anim Hosp Assoc. (2018) 54:1–21. doi: 10.5326/JAAHA-MS-6822, PMID: 29314873

[21] Parkes JL Slatin SL Pardo S Ginsberg BH. A new consensus error grid to evaluate the clinical significance of inaccuracies in the measurement of blood glucose. Diabetes Care. (2000) 23:1143–8. doi: 10.2337/diacare.23.8.1143, PMID: 10937512

[22] Pfützner A Klonoff DC Pardo S Parkes JL. Technical aspects of the Parkes error grid. J Diabetes Sci Technol. (2013) 7:1275–81. doi: 10.1177/193229681300700517 24124954 PMC3876371

[23] Tardo AM Irace C Del Baldo F Foglia A Fracassi F. Clinical use of a 180-day implantable glucose monitoring system in dogs with diabetes mellitus: a case series. Animals. (2022) 12:860. doi: 10.3390/ani12070860 35405848 PMC8996934

[24] Kristensen GB Christensen NG Thue G Sandberg S. Between-lot variation in external quality assessment of glucose: clinical importance and effect on participant performance evaluation. Clin Chem. (2005) 51:1632–6. doi: 10.1373/clinchem.2005.049080, PMID: 16120948

[25] Kimberly MM Vesper HW Caudill SP Ethridge SF Archibold E Porter KH. Variability among five over-the-counter blood glucose monitors. Clin Chim Acta. (2006) 364:292–7. doi: 10.1016/j.cca.2005.07.027, PMID: 16143321

[26] Paul AE Shiel RE Juvet F Mooney CT Mansfield CS. Effect of hematocrit on accuracy of two point-of-care glucometers for use in dogs. Am J Vet Res. (2011) 72:1204–8. doi: 10.2460/ajvr.72.9.1204, PMID: 21879978

